# Internalization of subcellular-scale microfabricated chips by healthy and cancer cells

**DOI:** 10.1371/journal.pone.0194712

**Published:** 2018-03-30

**Authors:** Kokab B. Parizi, Demir Akin, H.-S. Philip Wong

**Affiliations:** 1 Department of Electrical Engineering, School of Engineering, Stanford University, Stanford, California, United States of America; 2 Center for Cancer Nanotechnology Excellence, Department of Radiology, School of Medicine, Stanford University, Stanford, California, United States of America; University of Colorado Denver School of Medicine, UNITED STATES

## Abstract

Continuous monitoring of physiological parameters inside a living cell will lead to major advances in our understanding of biology and complex diseases, such as cancer. It also enables the development of new medical diagnostics and therapeutics. Progress in nanofabrication and wireless communication has opened up the potential of making a wireless chip small enough that it can be wholly inserted into a living cell. To investigate how such chips could be internalized into various types of living single cells and how this process might affect cells’ physiology, we designed and fabricated a series of multilayered micron-scale tag structures with different sizes as potential RFID (Radio Frequency IDentification) cell trackers. While the present structures are test structures that do not resonate, the tags that do resonate have similar structure from device fabrication, material properties, and device size point of view. The structures are in four different sizes, the largest with the lateral dimension of 9 μm × 21 μm. The thickness for these structures is kept constant at 1.5 μm. We demonstrate successful delivery of our fabricated chips into various types of living cells, such as melanoma skin cancer, breast cancer, colon cancer and healthy/normal fibroblast skin cells. To our surprise, we observed a remarkable internalization rate difference between each cell type; the uptake rate was faster for more aggressive cancer cells than the normal/healthy cells. Cell viability before and after tag cellular internalization and persistence of the internalized tags have also been recorded over the course of five days of incubation. These results establish the foundations of the possibility of long term, wireless, intracellular physiological signal monitoring.

## Introduction

New tools that can continuously monitor in vivo biological processes inside a living cell can not only answer fundamental questions on normal cell biology but also answer questions related to pathological biological processes involved in the development of diseases such as cancers. This type of detection capability could offer us novel early medical diagnostic tools and extended critical time for the treatments. However, current technologies do not yet provide non-optical real time access to the cell interior environment [1]. They provide either indirect access such as in optical/fluorescence imaging [2–5], which mostly requires labeling prior to the imaging or direct access albeit with cellular/tissue damage such as the invasive needle biopsy technique [6–8]. A miniaturized autonomous implantable sensor inside a cell that can track certain cellular changes such as pH, oxygenation, free radicals, or concentration of signaling proteins provides real time and non-invasive access to the cell intracellular environmental conditions and state of the cell [9]. Recent studies show that silicon-based particles with diameters ranging up to a few micrometers can be internalized by the living cells [10–17]. At the same time, progress in wireless communication [18,19], and integrated circuit (IC) fabrication techniques [20,21], opens the door for implementing an implantable silicon based RFID sensor chip small enough that the chip could be functional even inside the living cells. However, there are many challenges toward implementing a measurement system that detects the signal from a RFID sensor inside a cell. One of the key challenges is that the chip needs to be small enough so that it can be naturally internalized into the cell and the cell needs to continue to function after the internalization event. The transceiver for signal detection also needs to be close enough to the internalized tag in order to effectively detect the RF signal. For a tag with dimensions of the order of 20 μm, the RF frequency is in the 45 GHz range, as discussed in our previous work [18], where a 20 μm x 20 μm x 1.6 μm tag, coupled with a two-inductor transceiver, showed a sharp resonance peak in on-wafer measurements. Detailed analysis shows that the detection of this 45 GHz signal is limited to a range of several microns. Ongoing research is being carried out on internalizing the functional RFID tag structures into the target cell lines [22] and creating a detector platform that integrates microfluidic channels upon which transceiver units will be placed in the proximity of single cells with the internalized tag to facilitate detection. As a key component of this ongoing research, in this study, we investigated the required size and shape of the silicon chip that can be naturally delivered into the various types of living cell.

While there have been some studies on the effect of size and geometry of the target particles on efficiency of the internalization by macrophage cells [23–25], the particles that were used in these studies were made of special polymers which make the results not applicable to silicon based electronic devices fabricated using IC microfabrication techniques. Furthermore, the macrophage cells naturally phagocytose large particles, like bacteria, cell debris or even cancer cells, therefore the results are not relevant for epithelial cell lines that are non-macrophagocytic. There have been a few studies on internalization of silicon chip into the human HeLa cells (a non-macrophage cell type) using lipofection technique [11,12]. However, the silicon chips used in these studies are quite small and their sizes are not applicable for the functional RFID chip applications. We designed and fabricated a series of silicon-based micrometer tag structures to investigate the chip internalization requirements in terms of sizes and shapes of the chips so that they could naturally enter into the living cells efficiently and harmlessly. It is also notable that we have not applied any additional treatment to our silicon chips such as lipofection or special functionalization to deliver them into the cells. In order to understand how these internalized tags may affect cellular physiological parameters, we also performed experiments to monitor the cellular entry process, cell viability and activity before and after internalization of tags into the cells. We compared our observations among various types of living cells such as cancer and healthy cells. While the studied tag structure is not functional inside the cell, these results are important steps towards realizing the vision of cellular wireless sensors.

## Materials and methods

### Tag design, fabrication and extraction

**Fig 1:**
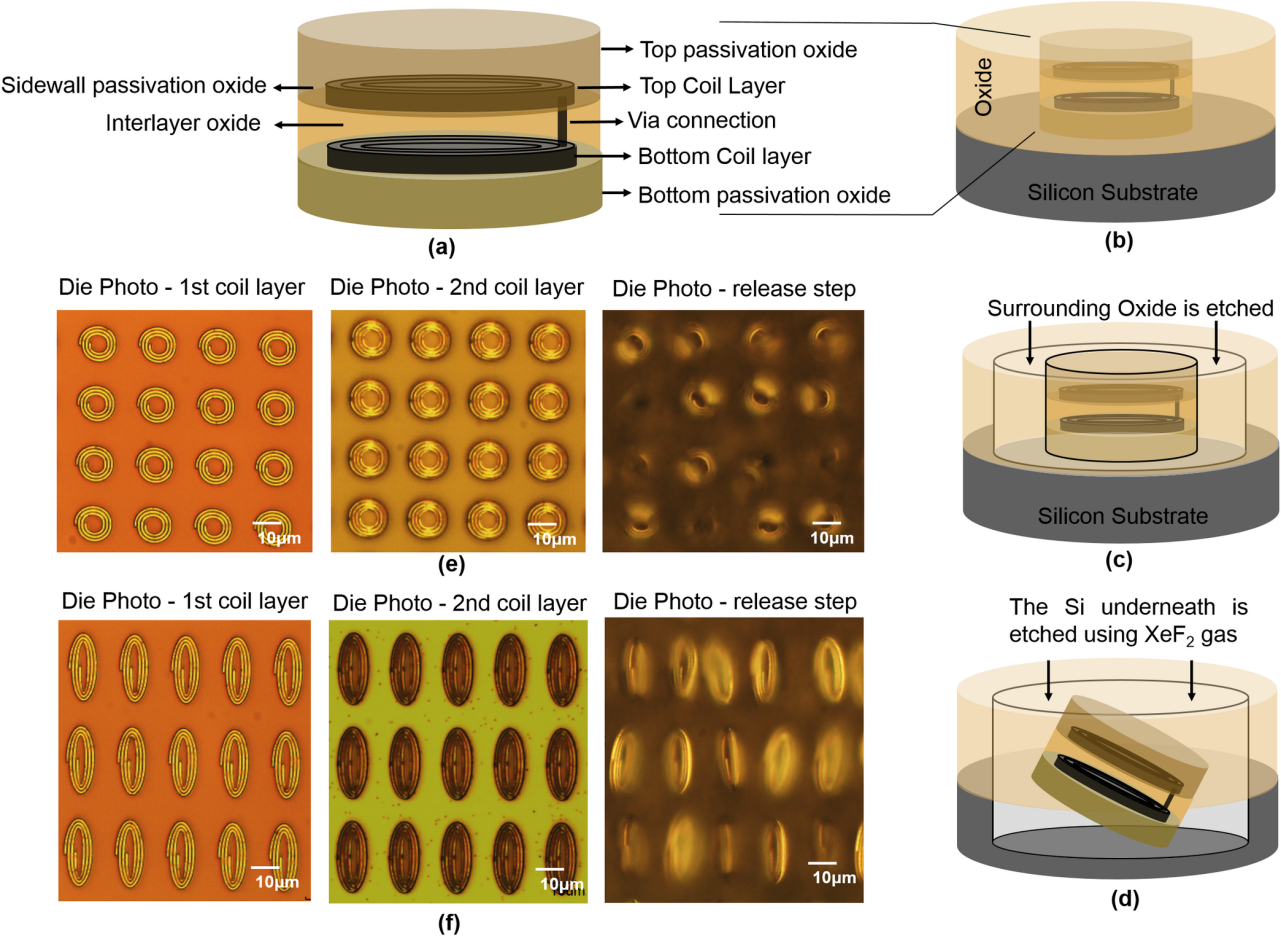
Fabrication, releasing process and the die photos of the tag structures at different stages of fabrication are illustrated. (a) The schematic of the tag structure, (b-d) the steps of the releasing process and the die photos of the structure for two tag sizes, (e) 12 μm × 12 μm and (f) 9 μm × 21 μm at different stages of fabrication, from left to right: after patterning the first coil layer, after patterning the second coil layer, and releasing step are shown.

The tags were designed with passive components, two layers of spiral coils (representing the inductor, L, of a resonant circuit) connecting at one end and folded on each other with oxide passivation layer in between which forms the capacitance (C) in this structure (Fig 1A). The tags were fabricated on silicon wafer using standard integrated circuit (IC) fabrication techniques. The fabrication of the structures started with growing the bottom encapsulation SiO2 layer on silicon wafer using thermal wet oxidation. The thermal wet oxidation of silicon was performed at temperature of 1000 °C and water vapor is used as the oxidant. The two inductor layers were made with 200 nm Al metal which was deposited via metal sputtering method. The 0.7 μm i-line SPR 955 CM resist was used for patterning the coil layers. The ASML (5500/60 model) 5:1 reducing stepper was used for UV exposure. The exposure energy was set to 90 mJ/cm^2^ to clearly define the 500 nm width patterns. The coil metal traces were formed by Al dry etching method. The dielectric between the inductors was silicon oxide (SiO_2_) with the thickness of 100 nm to provide sufficient isolation and also enough capacitance in the structure. The SiO_2_ dielectric was deposited using low-pressure chemical vapor deposition (LPCVD) and was patterned using photolithography and dry etching to expose the vias to connect the second coil layer to the bottom coil layer. A 500 nm thick LPCVD SiO2 was used to encapsulate the tags around the bottom, top, and sidewalls of the structure. The LPCVD oxide depositions were done by using the chemical reaction of the dichlorosilane (SiCl_2_H_2_) and nitrous oxide (N_2_O) in sub-atmospheric pressures. The tags were designed in four different sizes, the lateral dimensions ranges from 12 μm × 12 μm, 9 μm × 15 μm, 9 μm × 18 μm and 9 μm × 21 μm. The thickness for these structures was kept constant at about 1.5 μm. The different latteral dimensions were selected to monitor the effect of chip area and shapes of the tag structures for internalization study.

To release the tag structures from the substrate, the oxide around the tags was etched all the way to expose the Si substrate. The 1.6 μm Shipley-3612 resist and ASML exposure energy of 80 mJ/cm^2^ were used for patterning the surrounding oxide. The Xactix e-1 dry etcher tool with Xenon difluoride (XeF_2_) gas with gas pressure of 4 mTor was used for releasing the structures from the substrate. XeF_2_ gas isotropically etches the Si surrounding and underneath the tags until the tags separated from the silicon wafer. The fabrication and releasing process, and the die photos of the structures at different stages of fabrication are shown in Fig 1. To collect the tags from the wafer, the entire wafer with the released tags on top was rinsed with 50 mL of phosphate-buffered saline (PBS) for 5 times, the same solution was used at each time. The wafer was then placed horizontally at the bottom of a beaker with the PBS solution from the previous step on top. The beaker was placed in a VWR 50D Ultrasonic Cleaner for 4 min at power level 5. The solution with the suspended tags was then collected in a centrifuge tube for the cell delivery experiment. The tag concentration was about 1×10^4^ tags per mL which was then adjusted to higher concentrations by a centrifugation process based on the desired concentration for each experiment.

### Cells

Four different cell lines were used in this study; mouse melanocytic melanoma cells (B16-F10), human breast cancer cells (MCF-7), human colorectal cancer cells (HCT116) and human healthy fibroblastic primary cells (HS895.SK). The cells were obtained from the cell repository ATTC [26], which is a standard organization for preservation, development, and distribution of standard reference microorganisms and cell lines. The cells were kept in 25–75 cm^2^ cell culture flasks in a humidified incubator at 37°C and 5% CO_2_ and maintained in DMEM medium (Life Technologies) supplemented with 10% fetal bovine serum and 50 UI/ml penicillin, and 50 μg/mL streptomycin. A Countess II image-based cell counter (Life Technologies) was used for measuring cell population densities and trypan blue exclusion staining-based cell viability assessment. All of the microscopic observations and video microscopy recordings were performed on glass-bottomed chambered slides (8-well dishes, Fisher Scientific).

### Cell internalization and activity afterwards

Studying the delivery of the fabricated tag structure into the cells started by adding >85% viable cells at a concentration of approximately 1×10^5^ cells per mL in to the 8-well cell culture plates. Soon after that, the suspended tags in PBS were added to the cells with the tag to cell concentration of about 1 to 5. The cultured cells were then imaged continuously for several hours at different days of incubation using a Nikon scanning disk confocal (SDC) microscope. To maintain the cells in a suitable condition for several days, enough amount of DMEM cell medium was added to the dish and the cells were kept under the standard growth conditions (5% CO_2_, 37°C, humidified) for the entire time throughout the experiments. The Nikon SDC was used for both fluorescent and bright field microscopy. The Vybrant DiO cell-labeling solution [27], with the excitation and emission maxima at 484 nm and 501 nm, respectively was used for staining the lipophilic cell membranes for fluorescence microscopic observation. The DiO staining dye was added directly to normal culture media to uniformly label suspended or attached culture cells. The Vybrant DiD cell-labeling solution [28], with the excitation and emission maxima at 644 nm and 665 nm, respectively was used for staining the tag passivation oxide (unpublished observation). The DiD staining dye was added to the PBS solution containing the tags to uniformly stain the oxide passivation layer surrounding the tags. The tags were rinsed several times using PBS solution after staining to remove any excess dye that is not bound to the tag passivation layer.

### Biocompatibility check

The MTS based assay [29] was used for checking the biocompatibility of our tag structures. The 96-well plate format was used for maintaining the cells during our biocompatibility testing studies. The MTS assay is performed by adding a small amount of the CellTiter 96 AQueous One Solution Reagent (Promega) directly to the cultured cells and then recording absorbance at 490 nm with a 96-well plate reader (Molecular Dynamics, UV-Vis photo spectrometer scanner). The recorded absorbance measures the cell metabolic activity by the NAD(P)H-dependent cellular oxidoreductase enzyme reduction of a tetrazolium compound (3-(4,5-dimethylthiazol-2-yl)-2,5-diphenyltetrazolium bromide) with viable cells to generate a colored formazan product. The generated product is soluble in cell culture media and can be detected spectrophotometrically. For this purpose, each of the cell lines were tested with and without tags (the initial cell viability as determined by trypan blue staining was >85% in all experiments). The cells without-tag structures were used as the control samples. The initial cell concentrations were approximately 5x10^5^ cells per mL. The tag concentration was about 5 times lower in the wells with the presence of the tags. The MTS assay reagent was added to all cell culture dishes. For each combination, three separate replicates were used to provide additional statistical information. All cells were maintained for 72 hours without any passage. Representative samples were collected from each cell type at 4 consecutive interval times: shortly (within minutes) after incubating with the tags and then after 24 h, 48 h and 72 h of the incubation. The absorbance values of the collected samples at 490 nm wavelength are directly related to the cellular metabolic activity and biocompatibility levels. The collected samples at each day were stored in separate 96-well plates. The plates then were stored at -4°C fridge for future studies. After each measurement of the replicas, the rest of the test cells in wells were returned back to the cell culture incubator to continue provide the optimal condition for maintaining the cells.

### TEM sample preparation

The samples for transmission electron microscopy (TEM) were prepared by incubating the confluent culture of melanoma cancer cells with the concentration of about 5 x 10^6^ in the presence of our tag structures with the cell to tag ratio of 1:5 in an 8-well Lab-Tek chambered cover-glass dish. After 18 h incubation period under standard conditions (37°C, 5% CO2, humidified), the samples were prepared for TEM sectioning. The preparation procedure [17] started with fixing the samples with fixative solution at room temperature for about 40 minutes. The solution was made of 0.1 M Sodium Cacodylate (pH 7.4) with the same percentages of 2% glutaraldehyde and 4% p-formaldehyde. The samples were then fixed again in 1% osmium tetroxide solution. After rinsing the samples with distilled water, the samples were stained with uranyl acetate. The samples then were dehydrated in ethanol solution. Finally the samples filtered and embedded in Epon for manual sectioning with a diamond knife. The sections were then imaged with JEOL JEM1400 images using a Gatan Orius model 832.

### Statistical analysis

The data on tag internalization rate and tag biocompatibility are expressed as the mean±standard deviation (s.d.) as indicated with the error bars in presented graphs. The results on tag internalization rate are from about n = 1,000 initial cells from each cell types which then are incubated with the tags with the cells to tags ratio of 5:1. The data on cell biocompatibility are from m = 3 independent confluent cultures of each cell type with and also without tags.

## Results and discussion

In this study, the tag internalization event, the cellular uptake rate, and cell activities during the incubation such as persistence of the internalized tags inside the cells and the cell viability over time for each cell type have been monitored. The results of some of the experiments on these subjects including the images, videos of internalization event and cells activity after internalization are provided in the supplementary information.

### Melanoma skin cancer cell (B16-F10)

**Fig 2:**
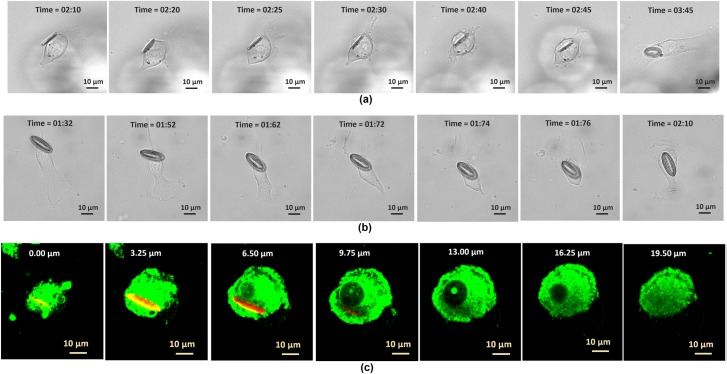
Internalization of fabricated tag structures into the melanoma skin cancer cells. The time sequence images of two internalization events for two tag sizes: (a) 9 μm × 15 μm and (b) 9 μm × 21 μm. (c) The Z-sectional images of the fluorescent labeled cells and tag with the depth of focus varies by 1 μm, the tag size is 9 μm × 18 μm.

In our experiment, a confluent culture of melanoma skin cancer cells were incubated with the tags. Fig 2A and 2B show two cellular uptake events of the tags with the lateral sizes of 9 μm × 15 μm and 9 μm × 21 μm, respectively. The bright field videos of these two internalization events are available in supplementary information S1 and S2 Videos. The internalization event under the fluorescent visualization mode is available in S3 Video. The way that the tags go inside the cells, the shape change of the cells right after taking the tags and the movements of the tags with the cells for several hours without them being dragged outside confirm that the individual tags were inside a given cell. The cross sectional TEM images of a cell with the tag at two separate sections (S1 Fig) further confirm that the tags are inside the cells. The cell with the tag inside was manually sectioned with a diamond knife. Since the tag structure is more rigid than the cell materials, some damage to the tag structure happened during manual sectioning. The TEM images also show some of the cell organelles inside the cell cytoplasm and therefore the location of the tag compared with these organelles are demonstrated in these figures. Furthermore, the confocal microscopy Z-sectional images, e.g. of a cell after internalization of a tag with the size of 9 μm × 18 μm with the depth of focus varying by 3.25 μm increments under the fluorescent visualization mode (Fig 2C), also confirm that the tags are inside the cell. The fluorescent stain DiO which was used for staining the cells, labels the cell lipophilic-structures, including the cell membrane, and it can be seen clearly that the tag is inside of these structures. The Z-sectional image sequences show that by increasing the stack height from Z = 0.0 μm to Z = 16.25 μm, the tag is starting to appear and then disappear when the stack height reaches beyond 9um and this is because the tag width is about 9um and the tag is located vertically on its bigger axis inside the cell. More detailed images about this Z-sectional image sequence are given in supplementary information S2 Fig. The video of this example cell Z-section is available in supplementary information S4 Video. While imaging under the fluorescent mode provides better views or accuracy, it was only limited to a few experiments due to the photo-damage that occurred from the laser excitation during the extended video microscopy observations of the cells by the confocal microscope [30]. Even with minimized laser energy and exposure time, the studied cells did not survive for longer than few hours during the imaging. Therefore, most of our results are obtained under the bright field mode as we have not seen any cell damage under the bright field mode for even much longer periods and much shorter time intervals during the video capture. The supplementary S5 Video shows the cell that has internalized a tag with the size of 9 μm × 21 μm went for successful cell division. This video confirms that the cell physiology including the cell division was not affected after taking the tags in.

**Fig 3:**
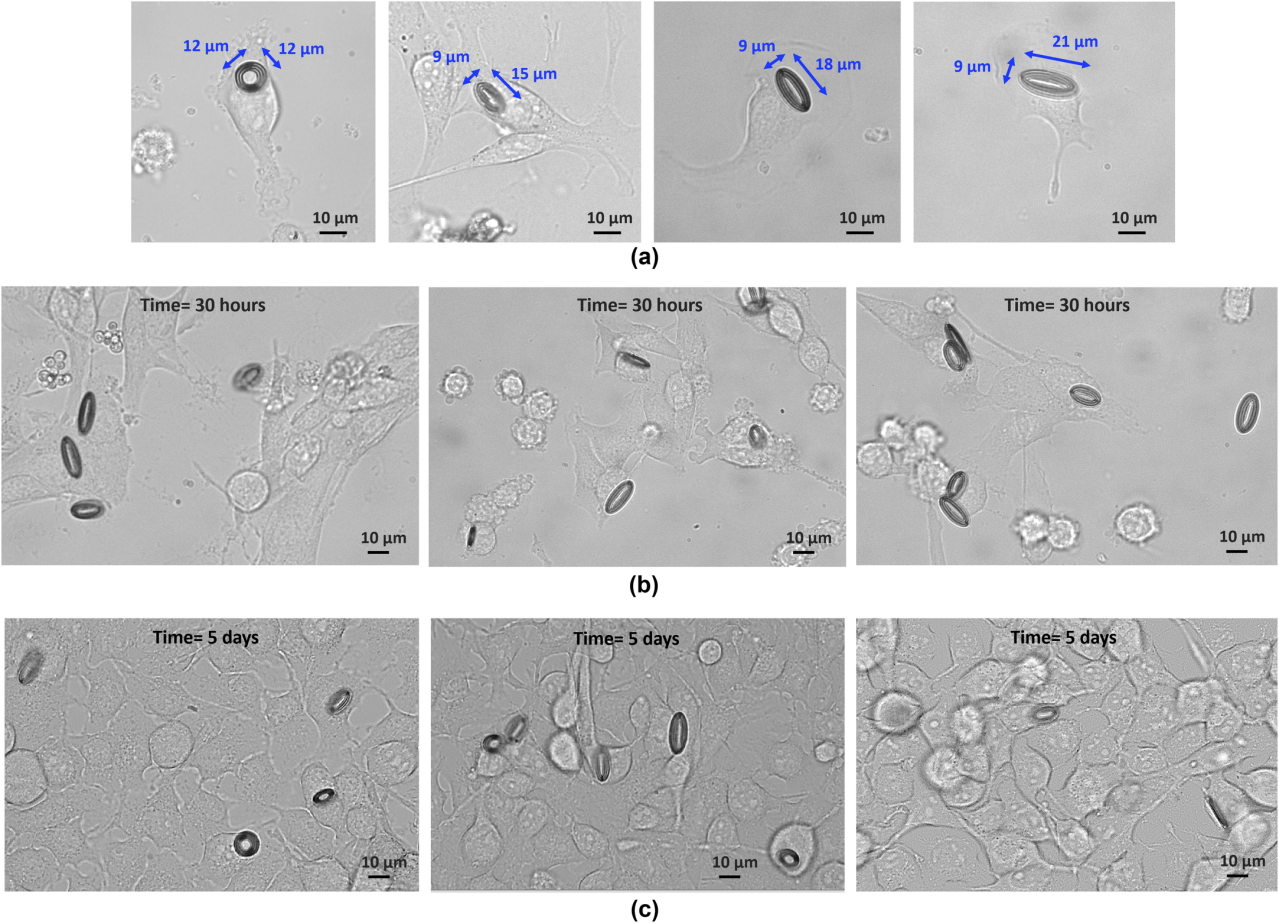
Melanoma skin cancer cells incubated with fabricated tag structures. (a) The bright field images of 4 different sizes of tags are delivered into the melanoma skin cancer cells. (b) The confluent culture of the melanoma cancer cells after 30 hours, and (c) 5 days of incubation with the tags.

The bright field images of the melanoma cells containing different sizes of tags are shown in Fig 3A. The snapshot images of the cells after 30 hours and 5 days of incubation with the tags are illustrated in Fig 3B and 3C respectively. The multiple images in each part are representative of different locations in studied cultured dish. In this experiment, similar internalization rates were seen from the tags regardless of their sizes. About 45% of the tags were seen taken up by the melanoma cells during the first 12 hours of incubation. The image at day 5 confirms the cell viability and the undisrupted growth of the cells over time. It also demonstrates the persistence of the tags inside the cells over the course of 5 days incubation. More details on internalization rate and cell viability are given later in this paper.

### Breast cancer cells (MCF-7)

Experiments similar to those carried out with the melanoma cancer cells were also done with the breast cancer cells. The S3 Fig demonstrates two internalization events of the tags into the breast cancer cells. The tags are in two different dimensions, 9 μm × 15 μm (S3A Fig) and 12 μm × 12 μm (S3B Fig). The time lapse video of both of these internalizations is available in S6 Video. It is also notable that the breast cancer cells were the most aggressive cell type in our study with respect to tag internalizing pace and doubling/growing rate. As it is seen in S7 Video, most of the tags are inside the cells even before starting the imaging which was shortly after adding the tags to the cell culture. In this video, it is also seen that a cell went through the successful cell division process while there were a tag inside it. The supplementary S8 Video shows the incubation of breast cancer cells with the tags for about 10 hours. As it is seen the breast cancer cells divide so fast and occupy most of the available cultured dish areas in a short time. Another successful cell division happened from a cell with the tag inside for a cell in upper left corner of this video. The snapshot images of the tags with different sizes taken by the breast cancer cells are illustrated in S4A Fig. The confluent cultures of the cells after 30 hours of incubation with the tags are also shown in S4B Fig. Some of the internalized tags had moved into the cell vacuoles [12, 31] within the first day of incubation (the cells indicated by the arrows in S4B Fig). This phenomenon coupled with a functional tag-based sensor can help to diagnose certain diseases which are related to the abnormal function of the subcellular compartments/vacuoles. The cell vacuoles destroy pathogens and can be dysfunctional with certain diseases. The formation of this vacuole/bubble shape structure that holds the tags is another confirmation that the tags are inside the cells. Similar to the results of the melanoma cancer cells, the uptake rate was quite similar for the tags regardless of their sizes. However, larger percentage of the tags (about 80%) was seen taken by the breast cancer cells during the first 12 hours of incubation. More statistical data on uptake rate is provided later in this paper.

### Colon cancer cells (HCT116)

The internalization study of the colon cancer cells was also included in this study to compare the cell uptake of our fabricated tags by various types of cancer cell lines. The time-lapse images of the colon cancer cells internalizing the tags with two different sizes, 9 μm × 15 μm and 9 μm × 21 μm are shown in S5A and S5B Fig, respectively. The videos of internalization are provided in supplementary S9 and S10 Videos. The bright field images of the tags with 4 different dimensions that were taken by the colon cancer cells are illustrated in S6A Fig. The snapshot images of the colon cancer cells after 30 hours of incubation with the tags are shown in S6B Fig. Similar to the breast cancer cells, some of the tags were seen in the cell vacuoles (the arrows in S6 Fig indicating the cell vacuoles). About 10% of the tags were seen internalized by the colon cancer cells during the first 12 hours of incubation. More statistical data on uptake rate is provided later in this paper.

### Healthy fibroblast cells (HS895.SK)

To compare the cellular uptake results obtained with various cancer cell lines with the normal healthy cells, we did similar experiments with healthy human fibroblast skin cells. Surprisingly, we observed the cell delivery of our tag structures into the normal cells as well. Supplementary S7 Fig shows the time lapsed images of the healthy cells internalizing the tag with the lateral sizes of 12 μm × 12 μm. The supplementary S11 Video shows the video of this internalization. S12 Video monitors another healthy cell after internalization of a tag with larger lateral dimension of 9 μm × 21 μm. The bright field images of the tags with different sizes were taken by the healthy fibroblast skin cells and the snapshot images of the cell culture after 30 hours of incubation with the tags are given in S8 Fig. However, in the case of normal cells, lower percentage of the tags, less than 5%, was seen internalized by the healthy skin fibroblasts during the first 12 hours of incubation. The snapshot images of the normal fibroblast cells after 48 hours (S9A Fig) and 5 days of incubation (S9B Fig) show most of the tags that had internalized into the cells were seen in the cell vacuoles at 48 hours of incubation. The time-lapse images of the cells after 5 days (S9C Fig), confirm the cell viability and persistence of the tags inside the cells over time. The time lapse videos of the internalized tags at day 5 of incubation are provided in S13, S14 and S15 Videos. The movements of the healthy cells while the tags are inside the cell vacuoles are illustrated in S14 and S15 Videos.

### Cellular uptake rate

**Fig 4:**
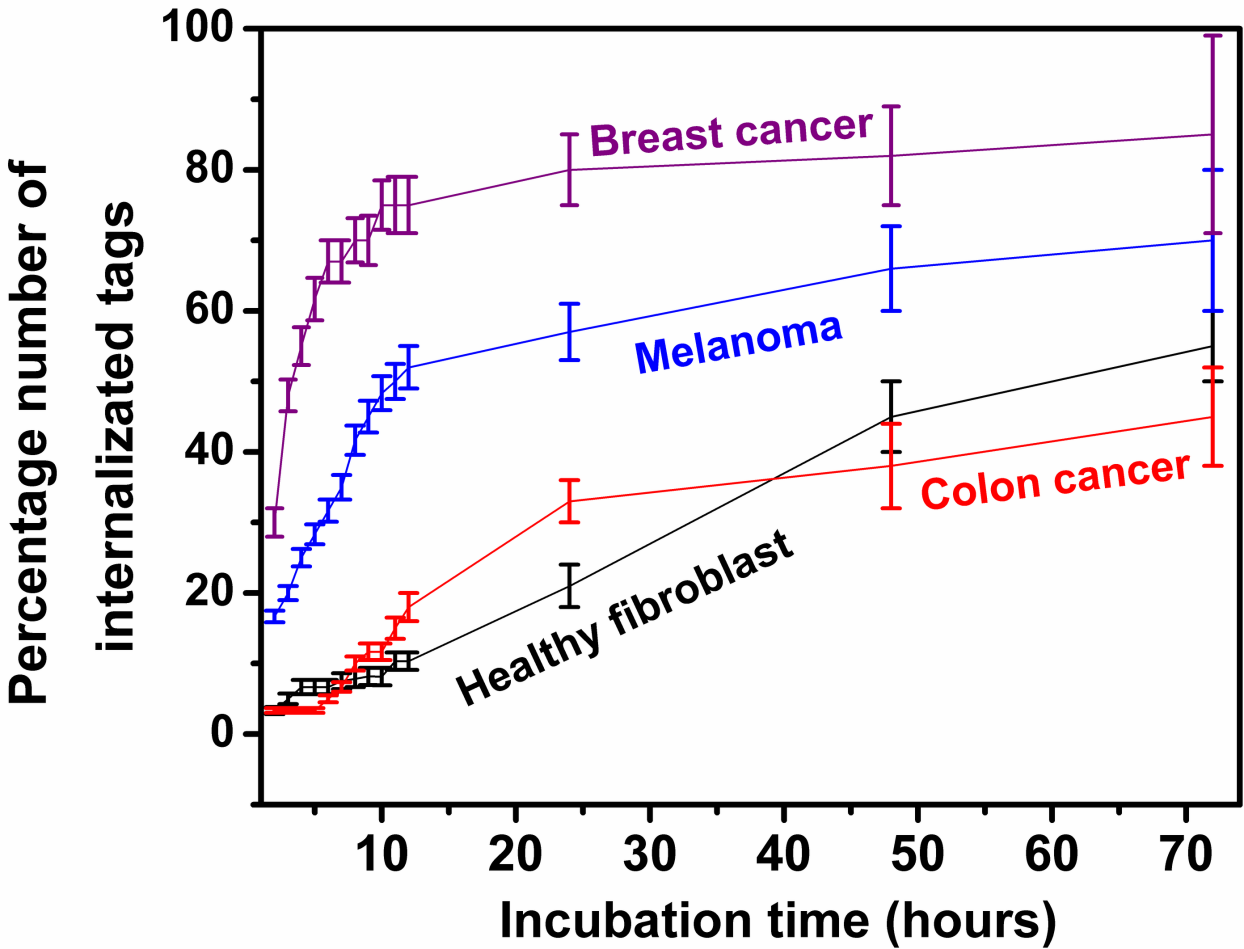
The percentage number of internalized tags into various types of living cells for 12 hours of cell tag incubation.

For each cell line, the activity of about 1,000 cells incubated with the tags with the initial cell to tag ratio concentration of about 5 to 1 were monitored for the first 72 hours after adding the tags into the cell culture. The first 12 hours of monitoring was continuously while the rest was at discrete times. The percentage of internalized tags over time is captured and shown in Fig 4. The tags in this graph are representative of all the four sizes that were used in this experiment as it was seen the number of internalizations over time was quite similar for the tags regardless of their sizes. As seen in Fig 4, each cell line demonstrates very different internalization rates over the observation time. About 75% of the tags were seen taken up by the breast cancer cells during the first 12 hours. The uptake percentage reduces to about 50% when the tags were incubated with the melanoma cancer cells. The colon cancer and normal/healthy cells were seen not taking the tags effectively right after the incubation. Only about 20% and less than 10% of the tags were seen inside the colon cancer and normal healthy cells respectively after 12 hours of incubation. However, our observation of the cell cultures at 30 hours and at 2 days of incubation show that at longer times most of the tags were seen inside the cells regardless of their lower internalization rate at the beginning. In this study, we were more interested in capturing the cell delivery rate at early stages of incubation, e.g. less than 12 hours, since in practical applications, the tags can easily be cleared from the systemic circulation via the reticuloendothelial system within the first few hours of parenteral administration and excreted by the hepatobiliary/enteric route within a day or so if they are not taken up by the cells. Another reason that we are not interested in capturing the cell internalization rate for longer time is the difference in doubling time between each cell lines which makes it difficult to maintain consistent parameters between each cell cultures.

### Cell viability

**Fig 5:**
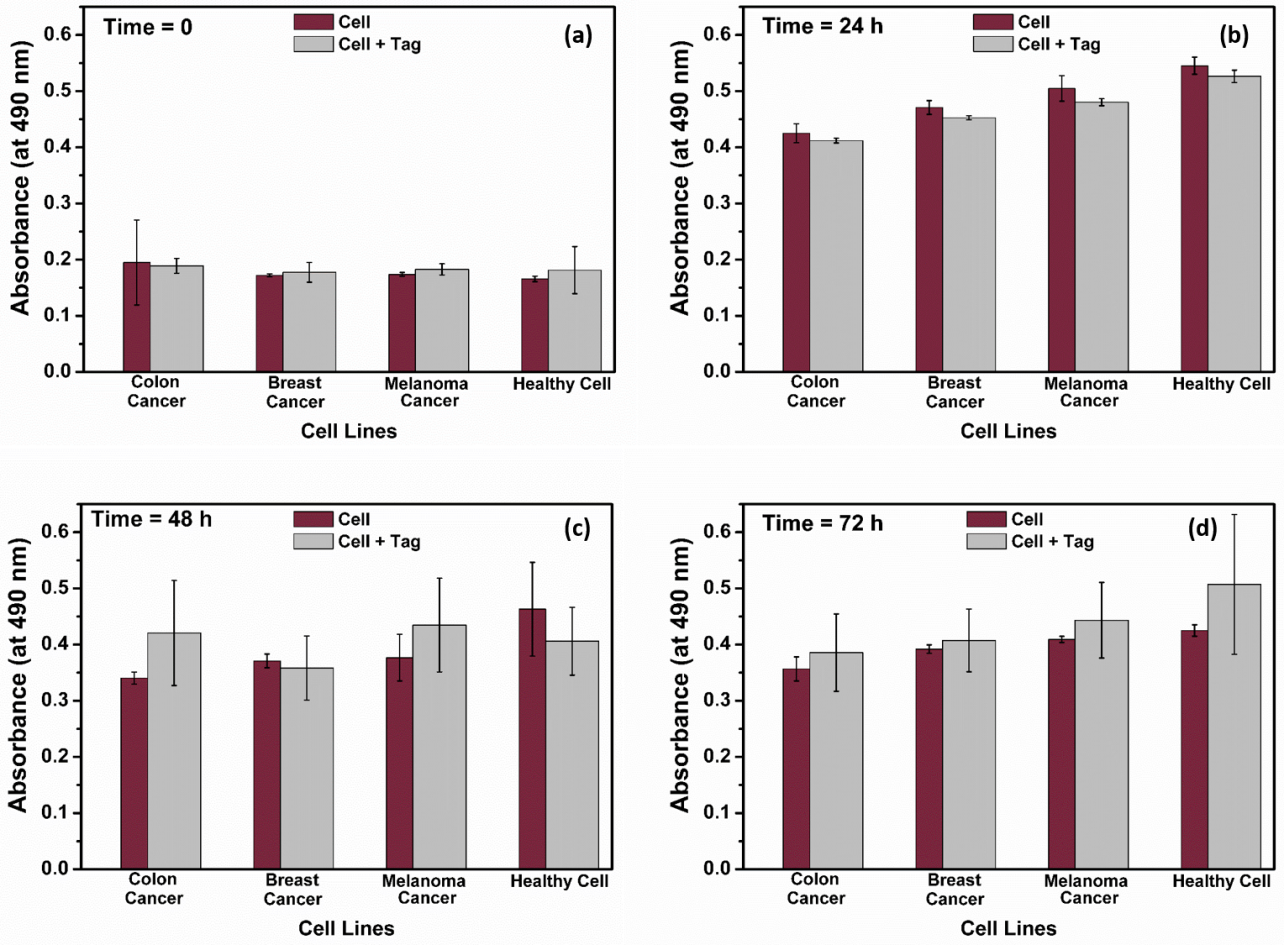
Cell viability and biocompatibility of the fabricated tag structure are confirmed in our study. Cell viability with and without the tags culture at (a) time = 0, (b) 24h, (c) 48h and (d) 72h were recorded by measuring the absorbance at 490 nm of the MTS-based assay.

An MTS based assay was used for testing the biocompatibility of the tags to the tested cells. The test was run according to the manufacturer’s standard instructions. In this experiment, the viability of two different cell types, with and without tags are monitored for 72 hours. The without-tag cultures were used as the control samples. The representative samples are from 4 consecutive times: shortly after incubating with the tags and then after 24 h, 48 h and 72 h of the incubation. The results are given in Fig 5 and it confirms the biocompatibility of our fabricated tag structure as no statistically significant differences in the cell viability rates were found between the cells with and without tags. The result from MTS assay for the cells with-tags depends on the viability of both cell groups of cells with and without tag inside. Since it is difficult to perform the experiments for only the cells with the tag inside, we can have an estimate on cell viability of the cells with the silicon chip inside by multiplying the initial cell to tag ratio and the tag internalization rate obtained from Fig 4.

## Conclusions

Herein, we have reported successful delivery of silicon chips with different lateral sizes (the largest ones have dimension of 9 μm × 21 μm) into various types of cancer and healthy cells such as melanoma skin cancer, breast cancer, colon cancer and healthy/normal fibroblast skin cells. We did not see any internalization rate differences between the studied tag structures with different sizes. It is also notable that we have not applied any special treatment to our silicon devices to deliver them into the cells. The cells naturally internalized the fabricated silicon chips within the first few hours of incubation. The confocal microscopic Z-sections of internalized tags as well as the cell-shape changes after internalization obtained from confocal fluorescent microscopy images confirmed that the tags are inside the cells. The tag biocompatibility and internalization rates by the studied cell lines were recorded. The cell viability and persistence of the tags inside the cell over time were also monitored over the course of 5 days of incubation. This work is an encouraging milestone towards realizing the vision for a new type of intracellular wireless biological sensors. In the near future, silicon-based implantable wireless sensors may allow real-time minimally-invasive monitoring of cellular activities at the level of single cells and pave the way for realization of intracellular medical nanorobotics as well as early detection of diseases at single cell levels.

## Supporting information

S1 Video Time-lapse bright field video of internalization of a tag with the lateral sizes of 9 μm × 15 μm into the melanoma skin cancer cell. (AVI)

S2 Video Time-lapse bright field video of internalization of a tag with the lateral sizes sizes of 9 μm × 21μm into the melanoma skin cancer cell. (AVI)

S3 Video Time-lapse video of internalization of a tag with the lateral sizes of 9 μm × 15 μm into the melanoma skin cancer cell under the florescent visualization. (AVI)

S4 Video The video created from the cell Z-sectional images of a melanoma cell with the 9 μm × 18 μm tag inside under the fluorescent mode. (AVI)

S5 Video The successful cell division of a melanoma cancer cell with a 9 μm × 21 μm tag inside. (AVI)

S6 Video The time lapsed video of internalization process of the tags with the sizes of 9 μm × 15 μm and 12 μm × 12 μm into the breast cancer cells. (AVI)

S7 Video The time lapsed video of the breast cancer cells one hour after incubating with the tags. Most of the tags were already inside the cells before starting the imaging.
(AVI)

S8 Video The time lapsed video of the breast cancer cells incubating with the tags for about 10 hours. The fast grow rate and cell division of the cells are demonstrated.
(AVI)

S9 Video Time-lapsed bright field video of internalization of a tag with the lateral sizes of 9 μm × 15 μm into the colon cancer cell. (AVI)

S10 Video Time-lapsed bright field video of internalization of a tag with the lateral sizes of 9 μm × 21 μm into the colon cancer cell. (AVI)

S11 Video The time-lapsed bright field video of internalization event of a 12 μm × 12 μm tag into the healthy fibroblast skin cell. (AVI)

S12 Video The bright field video of healthy fibroblast cells after internalization of a tag with dimension of 9 μm × 21 μm. (AVI)

S13 Video The Time-lapsed bright field video of internalized tags inside the healthy fibroblast cell after 5 days of incubation. (AVI)

S14 Video The Time-lapsed bright field video of internalized tags inside the healthy fibroblast cell after 5 days of incubation. (AVI)

S15 Video The Time-lapsed bright field video of internalized tags inside the healthy fibroblast cell after 5 days of incubation. (AVI)

S1 Fig The sectional TEM images of a cell with the tag structure inside at two separate sections; some damage to the tag structure occurs due to the manual microsectioning process. (TIF)

S2 Fig The Z-sectional image sequences of the cell with the tag inside under fluorescent visualization. (a) the image sequences at different stage heights are given,(b) the volume Z-sectional of the cell with the tag at stage height of Z = 3.25um is shown.
(TIF)

S3 Fig The time lapsed images of two internalization process by the breast cancer cells. The tags lateral dimensions are (a) 9 μm × 15 μm and (b) 12 μm × 12 μm.
(TIF)

S4 Fig Snapshot bright field images of the fabricated tag structures incubated with the breast cancer cells. (a) The bright field images of the breast cancer cells containing 4 different sizes of tags. (b) The confluent culture of the breast cancer cells after 30 hours of incubation with the tags.
(TIF)

S5 Fig The time lapsed bright field images of two internalization process by the colon cancer cells. The tags lateral dimensions are (a) 9 μm × 15 μm and (b) 9 μm × 21 μm.
(TIF)

S6 Fig Snapshot bright field images of fabricated tag structures incubated with colon cancer cells. (a) The images of the internalized different sizes of tags into the colon cancer cells. (b) The confluent culture of the colon cancer cells after 30 hours of incubation with the tags.
(TIF)

S7 Fig The time lapsed images of internalization event by the normal/healthy fibroblast skin cells. The tags lateral dimensions are 12 μm × 12 μm.
(TIF)

S8 Fig Snapshot images of fabricated tag structures incubated with normal fibroblast skin cells. (a) The bright field images of 4 different sizes of tags inside the cells. (b) The confluent culture of the cells after 30 hours of incubation with the tags.
(TIF)

S9 Fig Snapshot images of fabricated tag structures incubated with normal fibroblast skin cells at various time sequences. (a) The images are taken at 48 hours of incubation. (b) The images are taken at 5 days of incubation. (c) The time lapsed images of the cells after 5 days of incubation.
(TIF)
