# Supplementary figures and images for: Internalization of subcellular-scale microfabricated chips by healthy and cancer cells
Source: PLoS One. 2018 Mar 30;13(3):e0194712. doi: 10.1371/journal.pone.0194712 (PMC5877870; doi:10.1371/journal.pone.0194712)

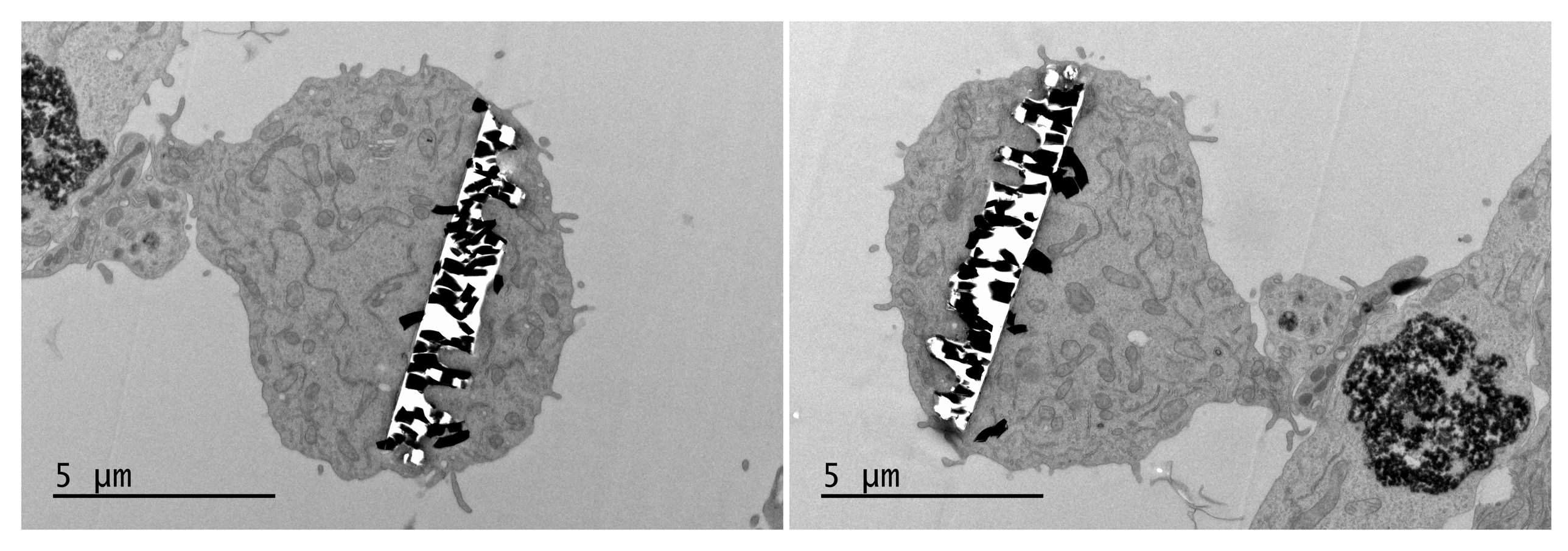

Supplement: S1 Fig — (TIF) [file pone.0194712.s016.tif]

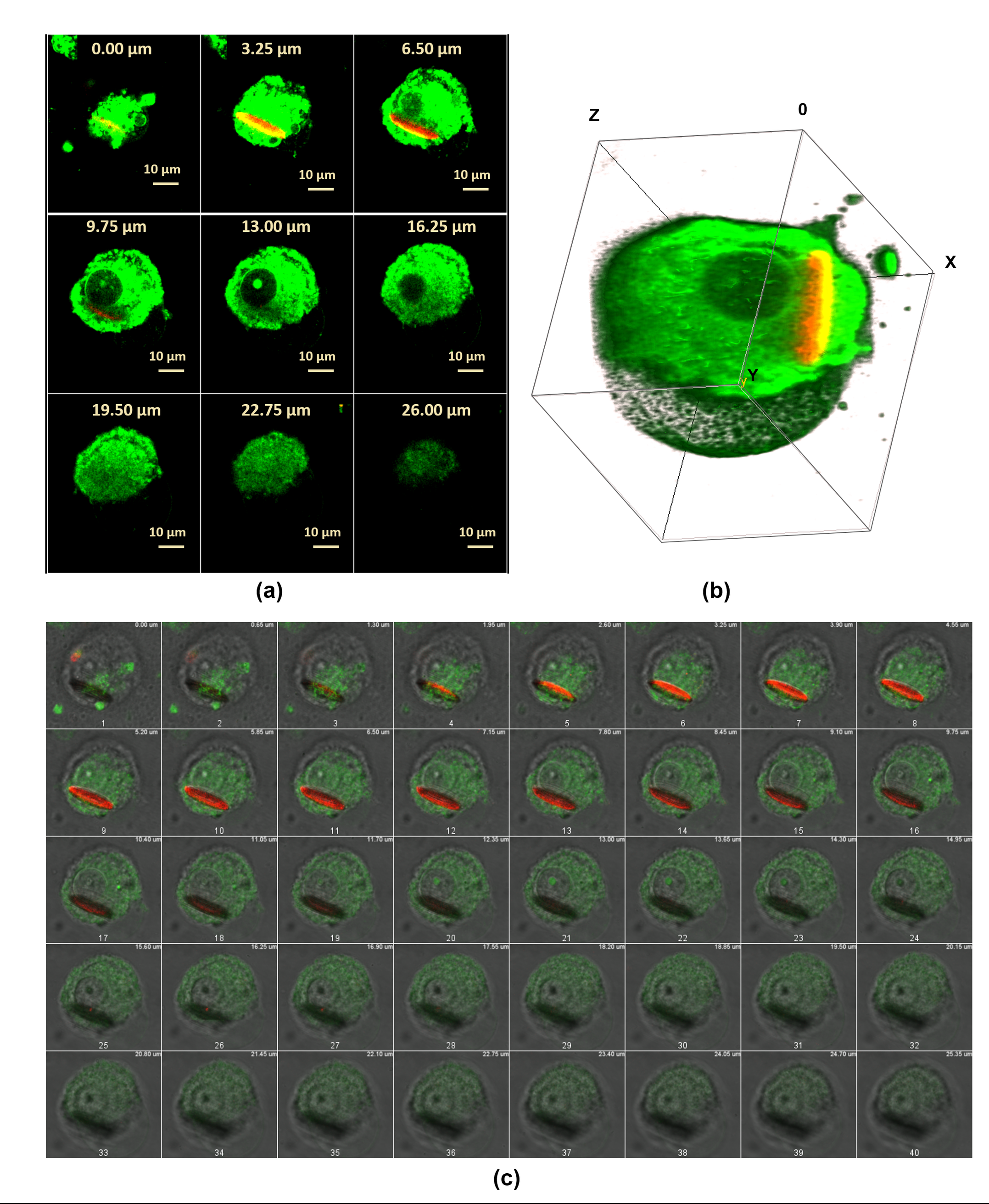

Supplement: S2 Fig — (a) the image sequences at different stage heights are given,(b) the volume Z-sectional of the cell with the tag at stage height of Z = 3.25um is shown. (TIF) [file pone.0194712.s017.tif]

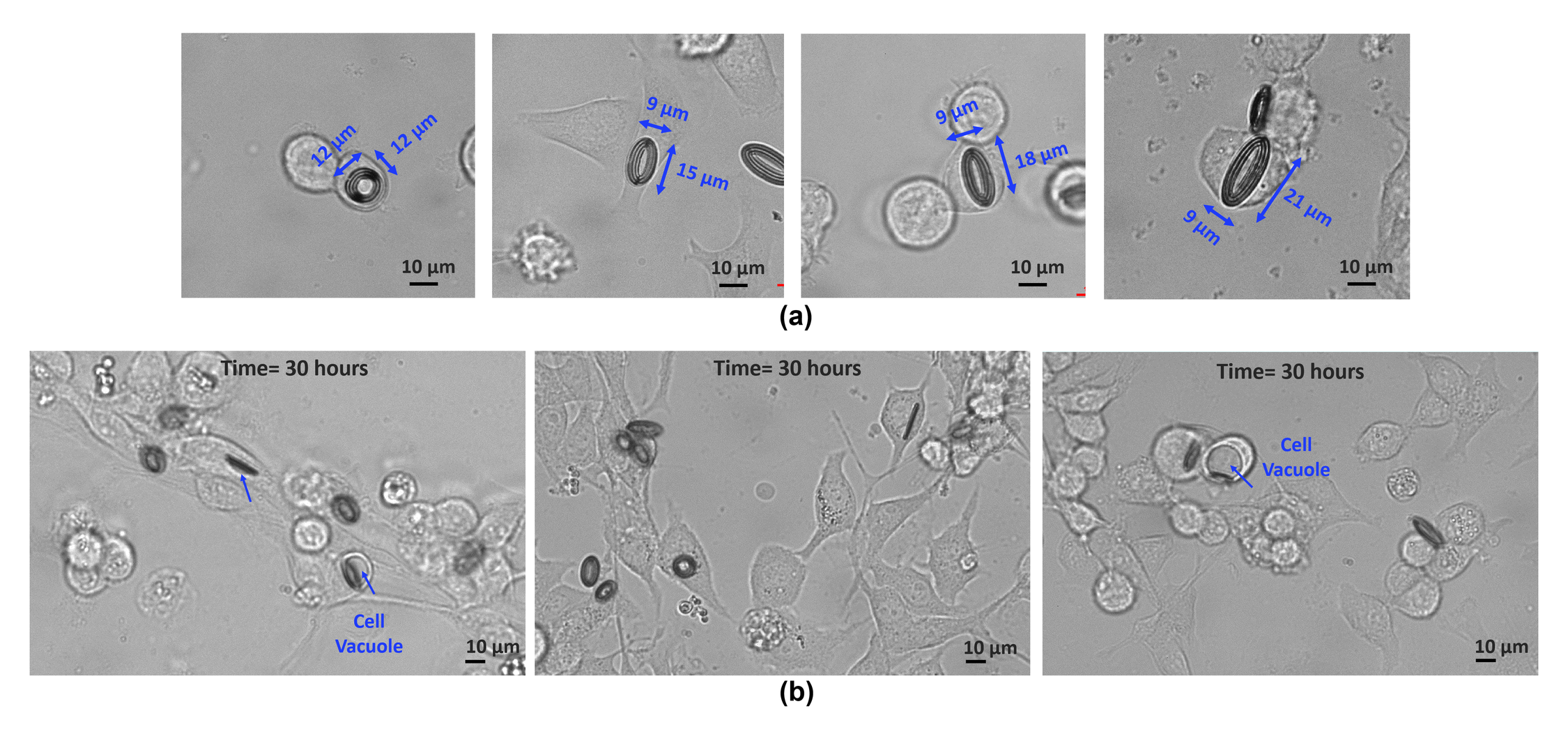

Supplement: S3 Fig — The tags lateral dimensions are (a) 9 μm × 15 μm and (b) 12 μm × 12 μm. (TIF) [file pone.0194712.s018.tif]

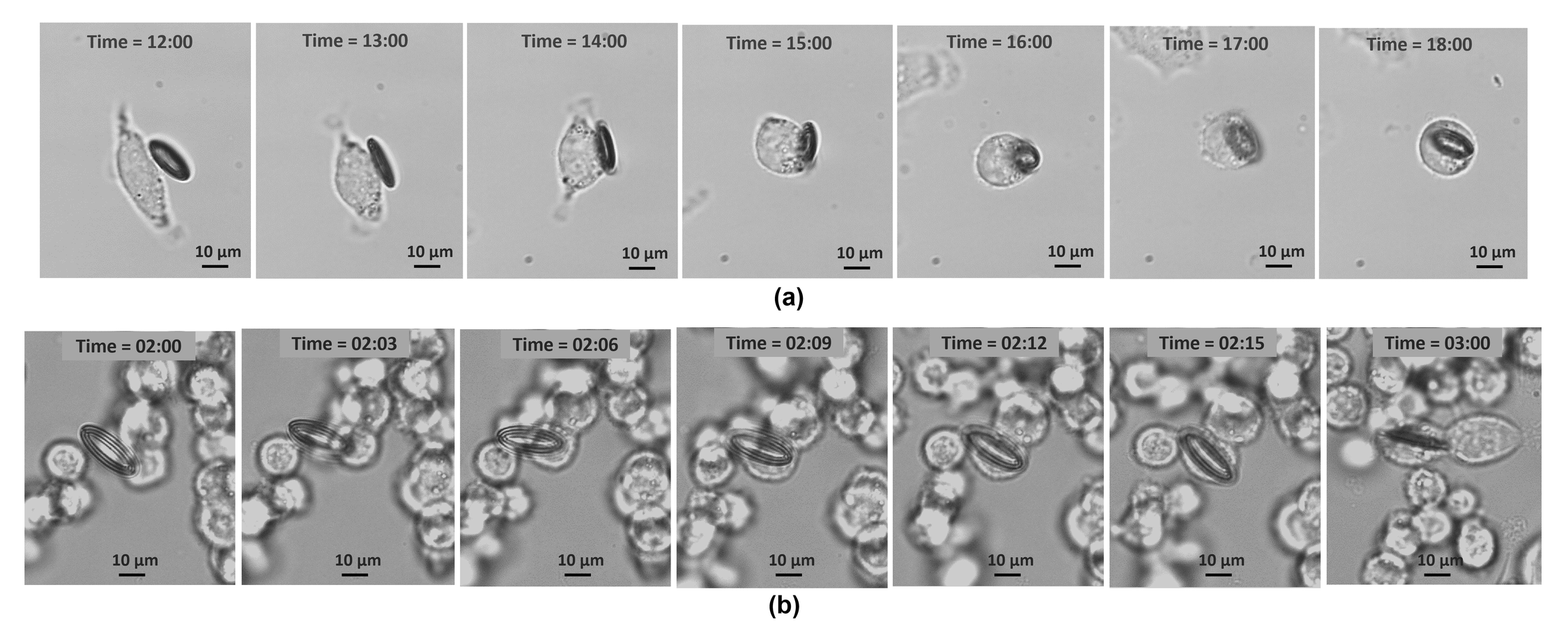

Supplement: S4 Fig — (a) The bright field images of the breast cancer cells containing 4 different sizes of tags. (b) The confluent culture of the breast cancer cells after 30 hours of incubation with the tags. (TIF) [file pone.0194712.s019.tif]

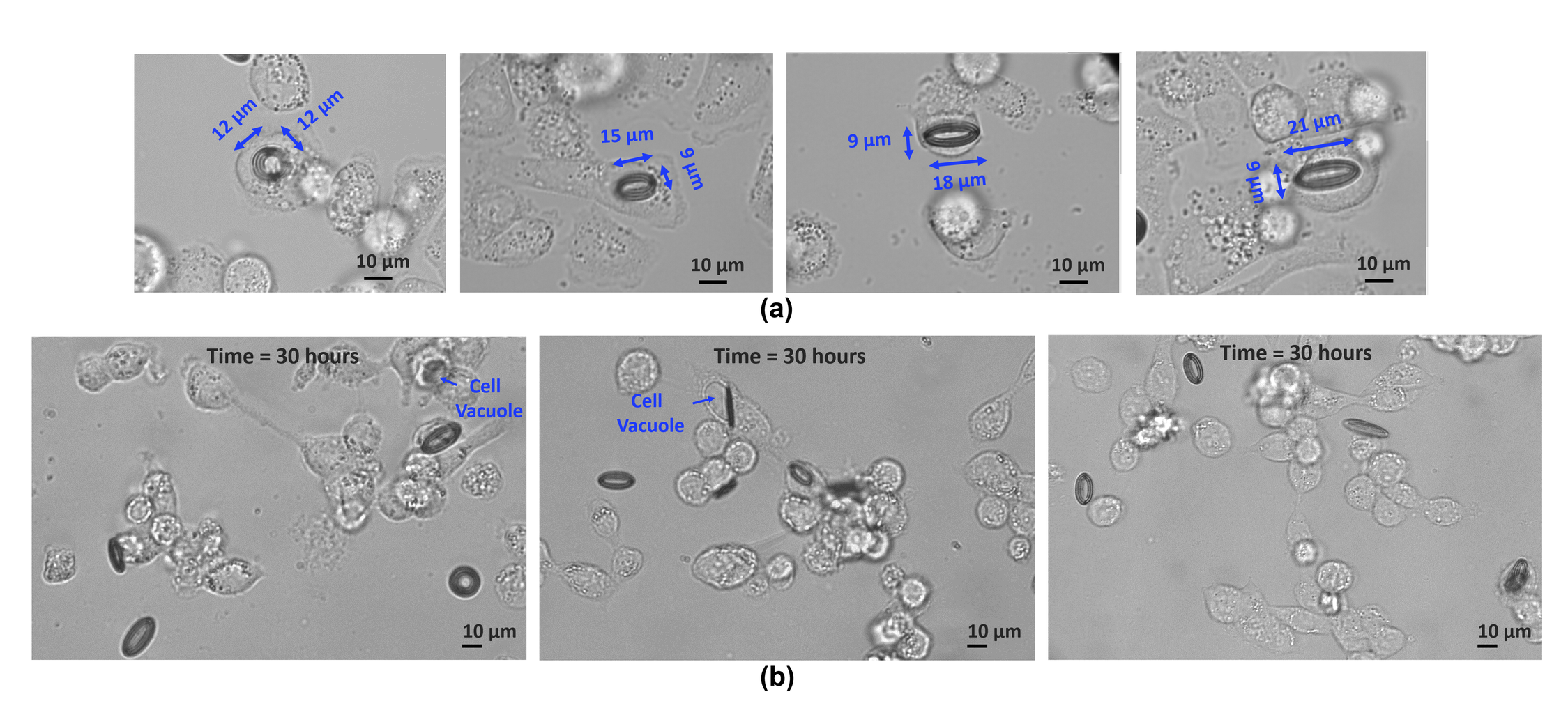

Supplement: S5 Fig — The tags lateral dimensions are (a) 9 μm × 15 μm and (b) 9 μm × 21 μm. (TIF) [file pone.0194712.s020.tif]

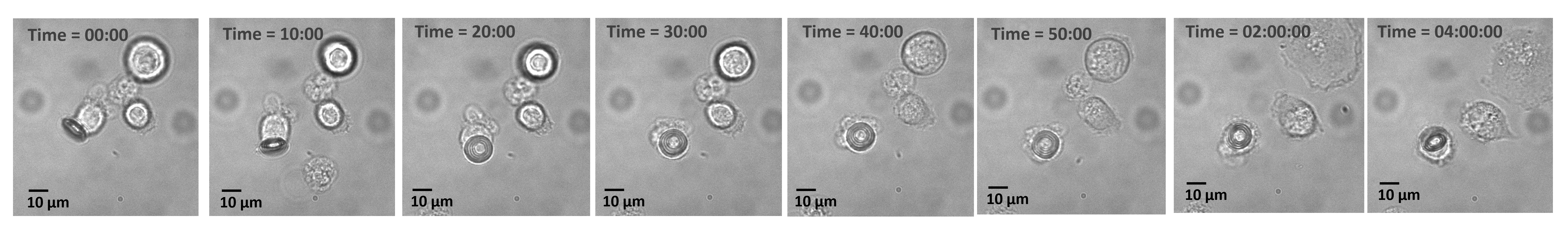

Supplement: S6 Fig — (a) The images of the internalized different sizes of tags into the colon cancer cells. (b) The confluent culture of the colon cancer cells after 30 hours of incubation with the tags. (TIF) [file pone.0194712.s021.tif]

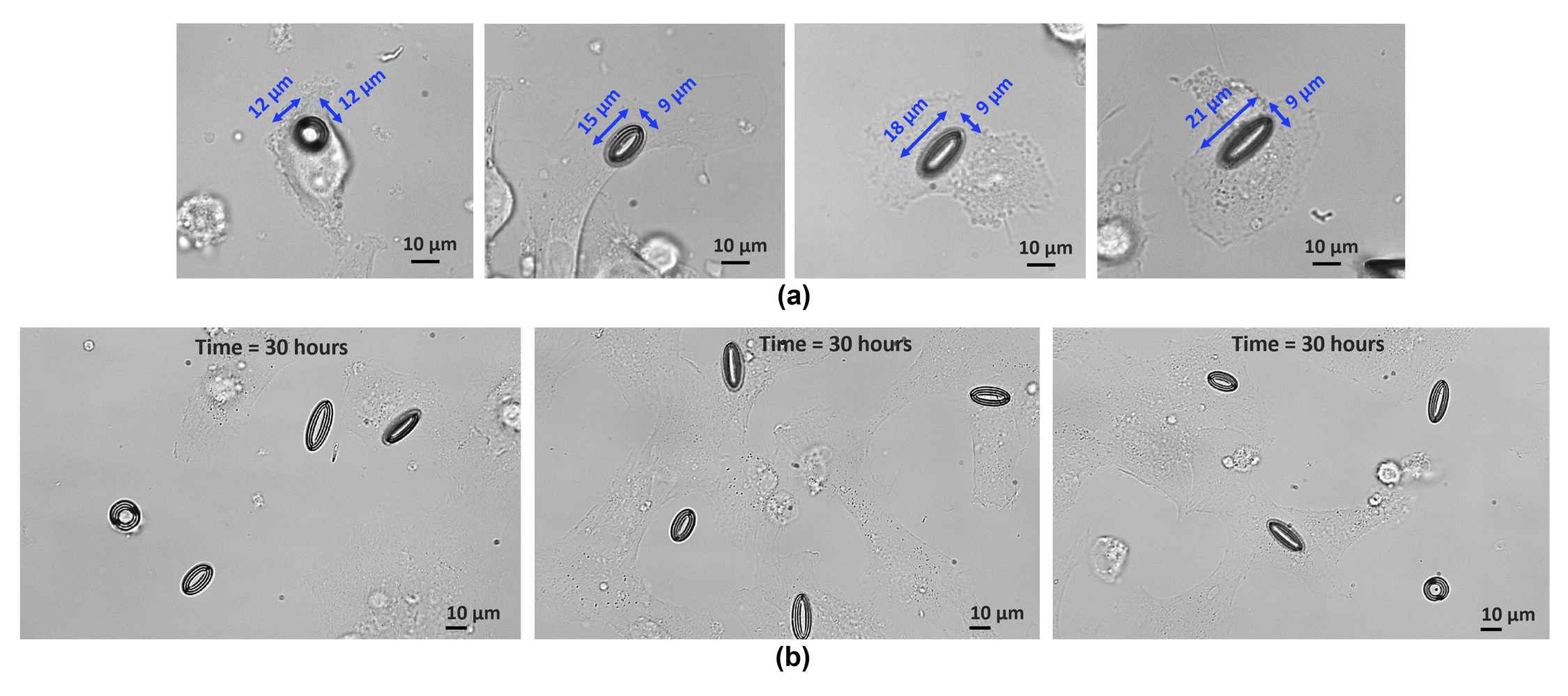

Supplement: S7 Fig — The tags lateral dimensions are 12 μm × 12 μm. (TIF) [file pone.0194712.s022.tif]

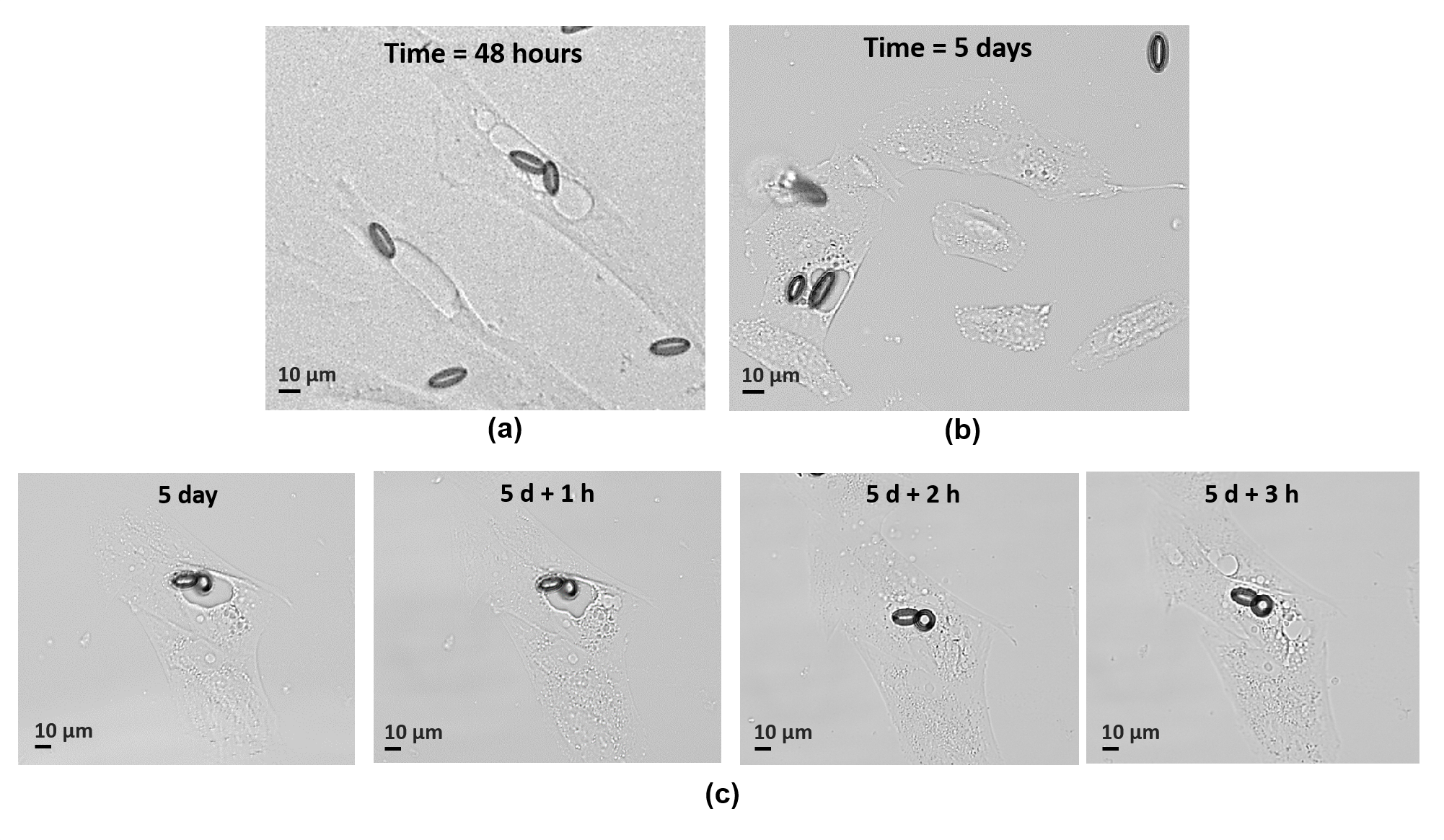

Supplement: S8 Fig — (a) The bright field images of 4 different sizes of tags inside the cells. (b) The confluent culture of the cells after 30 hours of incubation with the tags. (TIF) [file pone.0194712.s023.tif]
